# HyperSeed: An End-to-End Method to Process Hyperspectral Images of Seeds

**DOI:** 10.3390/s21248184

**Published:** 2021-12-08

**Authors:** Tian Gao, Anil Kumar Nalini Chandran, Puneet Paul, Harkamal Walia, Hongfeng Yu

**Affiliations:** 1 School of Computing, University of Nebraska-Lincoln, Lincoln, NE 68588, USA; tgao@huskers.unl.edu; 2 Department of Agronomy and Horticulture, University of Nebraska-Lincoln, Lincoln, NE 68583, USA; analinichandran2@unl.edu (A.K.N.C.); puneetpaul@unl.edu (P.P.); hwalia2@unl.edu (H.W.)

**Keywords:** hyperspectral imaging system, high-throughput seed phenotyping, phenotyping software, seed heat stress, 3D convolutional neural network (CNN), support vector machine (SVM), light gradient boosting machine (LightGBM), hyperspectral analysis

## Abstract

High-throughput, nondestructive, and precise measurement of seeds is critical for the evaluation of seed quality and the improvement of agricultural productions. To this end, we have developed a novel end-to-end platform named *HyperSeed* to provide hyperspectral information for seeds. As a test case, the hyperspectral images of rice seeds are obtained from a high-performance line-scan image spectrograph covering the spectral range from 600 to 1700 nm. The acquired images are processed via a graphical user interface (GUI)-based open-source software for background removal and seed segmentation. The output is generated in the form of a hyperspectral cube and curve for each seed. In our experiment, we presented the visual results of seed segmentation on different seed species. Moreover, we conducted a classification of seeds raised in heat stress and control environments using both traditional machine learning models and neural network models. The results show that the proposed 3D convolutional neural network (3D CNN) model has the highest accuracy, which is 97.5% in seed-based classification and 94.21% in pixel-based classification, compared to 80.0% in seed-based classification and 85.67% in seed-based classification from the support vector machine (SVM) model. Moreover, our pipeline enables systematic analysis of spectral curves and identification of wavelengths of biological interest.

## 1. Introduction

Seeds are essential for the modern agricultural industry since they are not only an important source for food supply but are also closely related to crop yield [1]. To obtain a precise quantitative evaluation of seed quality, plant scientists have studied various phenotyping methods. Traditionally, the seeds traits are measured manually. However, the manual measurement often involves error-prone, laborious, and time-consuming procedures for tackling massive number of seeds. Thus, there is a growing need for high-throughput phenotyping methods to generate a more precise quantitative measurement of seeds.

With the rapid development of sensors and computer vision technology, various methods have been presented for phenotyping. Based on 2D images captured by regular red–green–blue (RGB) cameras, researchers proposed various imaging systems [2,3,4,5]. Computer vision algorithms were also explored to obtain the traits of seeds. For example, Tanabata et al. proposed a software *SmartGrain* to obtain seed size and shape [6]. Zhu et al. developed an open-source software *SeedExtractor* for seed phenotyping using seed shape and color [7]. Besides RGB camera imaging, X-ray-based imaging has also proved a great potential for seed phenotyping. X-ray-based methods have been presented to obtain the structure of the seeds and to predict the germination capacity [8,9]. However, these methods have some limitations. For example, the X-ray assessment is usually time-consuming, potentially harmful, and not scalable for large-scale operations [10]. The methods based on RGB cameras can only capture the surface characteristics. Moreover, since RGB cameras only acquire three wavelengths (red, green, and blue), the spectral information on other wavelengths that potentially indicate the chemical composition traits are not measured [11].

To overcome some of these issues, plant scientists are exploring the hyperspectral imaging (HSI) technique that captures both spatial and spectral information. With a hyperspectral sensor, a wide range of wavelengths, including the ultraviolet (UV), visible (VIS), and near-infrared (NIR) spectra, can be obtained [12]. Due to its easy-to-operate advantages and scalability, HSI is becoming a popular tool to explore seed traits. For example, Wu et al. introduced an imaging system to determine seed viability by capturing the hyperspectral images from two sides of wheat seeds [13]. Hyperspectral imaging has also been applied to identify specific rice cultivars using deep learning techniques [14]. NIR hyperspectral images have been used to quantify the seed starch content [15].

Although various solutions have been developed for hyperspectral analysis of seeds, there are still unsolved problems. First, the existing software tools to process hyperspectral images are usually designed for general purposes rather than seed phenotyping. They are not directly suitable for tackling high-throughput phenotyping of seeds since they do not consider the unique features of seeds, such as shape. Second, the existing software tools developed by the vendors selling the hyperspectral cameras (e.g., Headwall and Middleton Spectral Vision) are proprietary, which makes it challenging to customize. Third, the cost of solutions, including imaging platforms and analytic software, is a limiting factor for many research laboratories in the public domain.

Moreover, climate change is driving the rising trend in temperature and posing a challenge for sustaining agricultural productivity. The mean annual temperature has increased by 1 °C for major regions in the past century [16]. The impact of high temperature has been well-documented for rice, which is estimated to suffer a 10% reduction in yield for every 1 °C increment in the growing season minimum temperature [17]. Besides yield, supranormal temperatures also decrease seed quality, as heat stress (HS) during the seed development phase drastically reduce the seed agronomic properties [18,19]. Poor seed quality under high temperature is a consequence of alteration in metabolic and transcriptomic signatures of seed development. For instance, phytohormones auxin, cytokinin, and ABA mediate the grain filling by regulating several genes that catalyze starch biosynthesis [20]. However, genes that fine-tune these biochemical pathways are impaired under HS [21]. A net effect of changes in the metabolic flux at the seed developmental phase leads to the production of low-quality seeds. Along with the reduction in seed size, abnormalities in grain filling pathways trigger distortion in the starch granule packing, thus rendering an opaque white appearance at the center of the endosperm, termed as grain chalkiness. While translucent grain fetches a maximum market price, chalky grains with low milling and cooking quality have less consumer acceptance [22]. The development of rice cultivars that can maintain seed quality under HS has become a key target for rice improvement. A similar scenario is just as pertinent for other major crops, which are relatively less characterized for heat sensitivity but are equally likely to suffer loss of yield and quality.

To address the challenges of HSI, we propose an end-to-end solution named *HyperSeed* that is designed for high-throughput seed phenotyping using HSI techniques. Moreover, to explore the rice seeds under HS and illustrate the application of *HyperSeed*, we also conducted an experiment as a case study. The proposed solution includes a lab-based imaging system coupled to an application with a graphical user interface (GUI) for hyperspectral analysis on seeds. The imaging system, which is cost-effective and easy to build, can capture the hyperspectral images of seeds at a large scale. The application is designed to process the images and extract the averaged hyperspectral reflectance for each seed in the form of comma-separated values (CSV) files that can be opened and viewed using conventional spreadsheet software tools. Hypercubes for each seed are generated for pixel-based analysis. This software removes the background, segments every single seed in an image, and calibrates the output. The general shape of seeds is considered in the process of seed segmentation so that the application can process seeds with overlapping regions, thus saving effort and time to spatially separate individual seeds. *HyperSeed* allows users to explore and modify parameters for better performance of their own datasets. Our experiments demonstrate that the system can be adapted to evaluate seeds from different plant species without any major modifications. Moreover, the application is implemented in MATLAB, which is open-source and available for researchers with an institutional license. For the users without a MATLAB license, we also provide a standalone version of *HyperSeed*, which only requires the free accessible MATLAB Compiler Runtime (MCR) for its operation.

In our case study on rice seeds, we performed an experiment for seed classification and hyperspectral analysis. We used heat-stressed rice seeds as the test case since the seeds developed under higher temperatures undergo morphological and biochemical changes that otherwise are challenging to quantify using manual methods. For this, two groups of rice seeds were utilized as samples; the seeds in the first group (control) were harvested from plants grown under a ambient temperature, while the seeds in the second group were harvested at maturity from plants exposed to a transient HS during seed development. Then, we applied a 3D convolutional neural network (3D CNN) [23] to classify the two groups and compared it with support vector machine (SVM) [24]. These popular supervised models differ in training sample generation and spatial information extraction. The experiment showed that the proposed 3D CNN achieved high accuracy in the classification of the two groups, probably by extracting the spectrum of neighboring pixels in the spatial direction. Moreover, we implemented a spectral analysis using a light gradient boosting machine (LightGBM) model [25]. The wavelengths of biological interest were identified.

## 2. Materials and Methods

**Figure 1:**
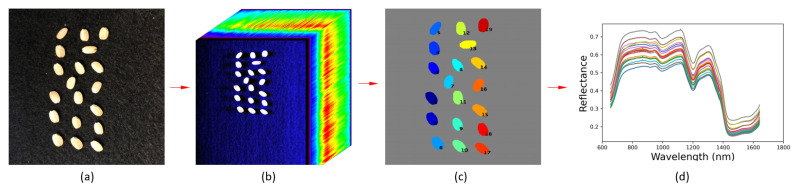
The overall workflow of proposed platform: (**a**) seed sample placement; (**b**) hypercube generation; (**c**) seed segmentation; (**d**) reflectance curves extraction.

The workflow of the proposed hyperspectral imaging system—*HyperSeed*—is illustrated in Figure 1. The seed samples (Figure 1a) are placed in the imaging system, and hyperspectral images are generated in the form of a hypercube (Figure 1b). The software processes the hypercube and segments each seed (Figure 1c). Finally, seed-based and pixel-based reflectance are extracted for further analysis. The seed-based reflectance is shown in Figure 1d, and each curve represents a single seed.

### 2.1. Sample Preparation

Seeds were dehusked and surface sterilized with bleach (40% *v*/*v*) for 40 min and soaked in sterile water overnight. The sterilized seeds were germinated for 2 days in dark and followed by 4 days in light on half-strength Murashige and Skoog media. Seedlings were then moved to the greenhouse in 4-inch (101.6 mm) pots filled with pasteurized soil. These plants were grown in the greenhouse under a diurnal condition with temperature 28/25 ± 2 °C, light/dark 16/8 h, and relative humidity of 55–60% until flowering. At flowering, open florets were marked for tracking the flowering time. Half of the plants at 1 day after the fertilization stage were moved and maintained in the high day and night temperature (HDNT) chamber (36/32 ± 2 °C) for 5 days to impose HS. HS-treated plants were then transferred to control conditions and grown until maturity. Mature and dehusked seeds from the control group and seeds of these HS-treated plants were used for HSI and further analysis.

### 2.2. Imaging System

**Figure 2:**
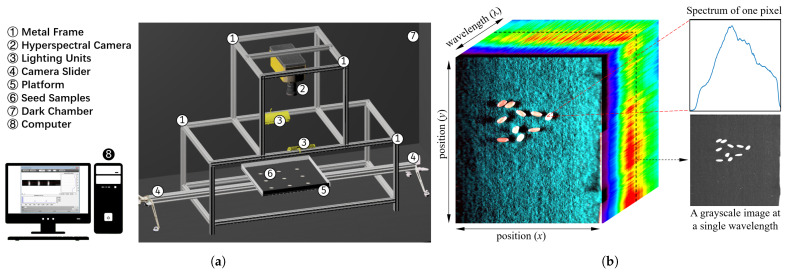
Hyperspectral imaging (HIS) system: (**a**) *HyperSeed* platform; (**b**) a generated hypercube of test-case seed samples.

Figure 2a demonstrates our imaging system. A metal frame is assembled using aluminum profile extrusion to hold the hyperspectral camera and the light source. A high-performance line-scan image spectrograph (Micro-Hyperspec${}^{®}$ Imaging Sensors, Extended VNIR version, Headwall Photonics, Fitchburg, MA, USA) is fixed on the top of the frame, which covers the spectral range from 600 to 1700 nm, with a 5.5 nm spectral resolution. The focal length and the minimum working distance of the camera lens are 25 mm and 300 mm, respectively. A two-line lighting unit with four 20 W tungsten–halogen bulbs for each line (MRC-920-029, MSV Series Illumination, Middleton Spectral Vision, Middleton, WI, USA) is fixed in the middle of the frame to illuminate the seed samples. The emission spectrum of the light source spans from 350 to 3000 nm. A camera slider with a track is placed on the bottom of the frame, and a square platform driven by a motor is installed on the track. Seed samples are placed on the platform and scanned line by line. The platform is painted black to reduce noise and facilitate the extraction of the seeds for downstream analysis. The whole system is placed in a dark chamber to eliminate external and varying light source. The chamber is installed in a room where the temperature and the humidity are controlled in the range of 66 to 74 °F, and 16% to 20%, respectively. A computer (Intel(R) Core (TM) i7-9700K CPU @ 3.60 GHz (Santa Clara, CA, USA), RAM 8 G) is located next to the dark chamber, and camera-controlling software (HyperSpec${}^{®}$III, Headwall Photonics, Fitchburg, MA, USA) is installed to set parameters for image acquisition.

### 2.3. Image Acquisition

Before image acquisition, the camera is turned on and warmed up to avoid baseline drift [26]. Subsequently, the controlling software developed by Headwall Photonics is used to adjust the parameters of the camera, such as exposure time, and take hyperspectral images. Other settings, such as the distance between the camera and the platform, are also calibrated to acquire the best quality images without distortion. For this study, the distance, exposure time, and frame period are set to 15 cm, 12 ms, and 18 ms, respectively. Rice samples are placed on the platform, and the image acquisition process is initiated. On average, it takes 15 s to capture one image, and the images are obtained in the form of three-dimensional (*x*, *y*, and λ) hypercubes. In this study, the hypercube includes 640 pixels in the *x* direction and 268 wavelength bands in the λ direction, respectively. The number of pixels in the *y* direction depends on the duration of imaging, and it varies from 1100 to 1600 in our experiment. As an example illustrated in Figure 2b, one captured hyperspectral image dataset in the form of a hypercube is shown in pseudocolor. A pixel on the *x*–*y* plane corresponds to a spectrum curve. A sliced image at a specific wavelength in the λ direction of the hypercube is shown in grayscale.

### 2.4. Software Implementation

**Figure 3:**
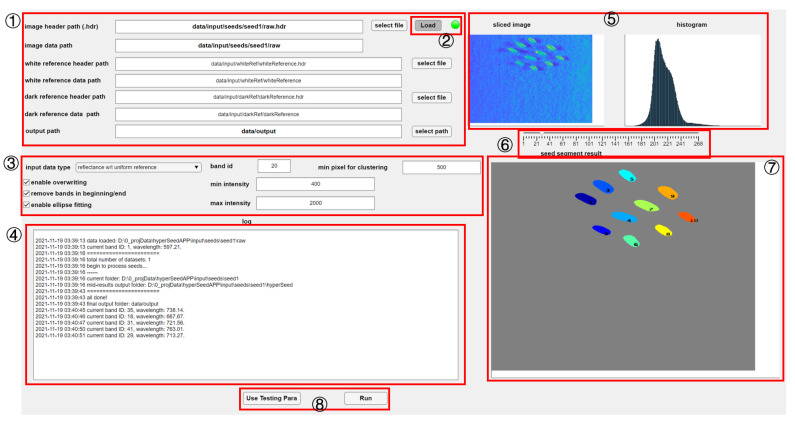
The GUI of *HyperSeed* software: (1) path region to define the path for input and output files; (2) loading region to load the dataset of interest for visualization; (3) setting region for parameter setting; (4) log region to display progress information; (5) visualization region for the sliced image and the corresponding histogram; (6) slider to specify wavelength for visualization; (7) visualization region for segmentation results; (8) buttons for initiating the image processing.

The *HyperSeed* software is utilized for the analysis of hyperspectral images. This open-source software is developed in MATLAB, and its standalone version can be operated without a MATLAB license. It has a GUI with multiple adjustable parameters for flexibility to process seed images shown in Figure 3. Users can process their seed samples following these steps: (1) path specification, (2) data visualization, (3) parameters setting, and (4) hypercube processing.

#### 2.4.1. Path Specification

*HyperSeed* application is compatible with hyperspectral images in the ENVI format consisting of pairs of raw images and header files. As shown in region 1 (Figure 3), the path of header files (*.hdr) and data filesof the hyperspectral image needs to be specified in *input data path*. *HyperSeed* is designed for batch processing, and a path with a regular expression is supported. As an example, [EXAMPLE PATH]/*.hdr loads all the hyperspectral images in the given path. Users also need to specify a path to output results in the *output path* box.

#### 2.4.2. Data Visualization

After specifying the data path, users can visualize the hypercube. By clicking the *Load* button in region 2 (Figure 3), a sliced 2D image is extracted and demonstrated in region 5 (Figure 3) for visualization. A slider under the image in region 6 (Figure 3) can be used to specify the wavelength for the visualized image. A corresponding histogram for image intensity is also generated and displayed on the right of the sliced image.

#### 2.4.3. Parameters Setting

A set of default parameters are automatically loaded when the application is launched. In region 3 (Figure 3), the checkbox *remove bands in beginning/end* is used to decide whether the software removes the bands at the beginning and end to increase accuracy. The checkbox *enable overwriting* decides whether the software overwrites the existing result files. The checkbox *enable ellipse fitting* decides whether an ellipse fitting algorithm, *RFOVE*, is included (Section 2.4.6). The dropdown box *input data type* controls whether the software generates intensity or reflectance. If intensity mode in the dropdown box is selected, only the path of hyperspectral images in region 1 (Figure 3) needs to be specified. Otherwise, if reflectance mode is selected, the paths of white and dark reference also need to be specified. The parameters of *band id*, *min intensity* and *max intensity* are utilized to create a mask for background removal. The parameter of *min pixel for clustering* decides the threshold for the minimal pixels of one seed in segmentation (Section 2.4.5).

#### 2.4.4. Hypercube Processing

Once all the parameters are set, the batch processing is ready to start. The application has no assumption about the position of the seeds, so that seeds do not need to be placed in the center. Moreover, results can be correctly generated even if there are overlapping regions between seeds. The users can start the batch processing by clicking the *Run* button in region 8 (Figure 3). The seed-based averaged reflectance and pixel-based reflectance are extracted if reflectance mode (Section 2.4.3) are selected in GUI. Otherwise, image intensity instead of reflectance is generated. All the results are sent to the *output path* for further analysis. The critical information, such as the total number of images and the path of current image being processed, is displayed in the log region, region 4 (Figure 3) for users to visualize the progress. In general, there are four main steps to process a hypercube: (1) initial seed segmentation, (2) refined seed segmentation, (3) spectral data extraction, and (4) image calibration. In the first two steps, we generate masks using image processing algorithms (Section 2.4.5 and Section 2.4.6) on a sliced image with *band id* specified in region 3 (Figure 3). In this work, various bands are tested, and band 20 is utilized due to the clear contrast between our seeds and the black background. The corresponding wavelength for band 20 is 675 nm. Then, the segmentation is achieved by utilizing the generated masks to the *x*–*y* plane of the hypercube. Segmentation results are visualized in region 7 (Figure 3).

#### 2.4.5. Initial Seed Segmentation

**Figure 4:**
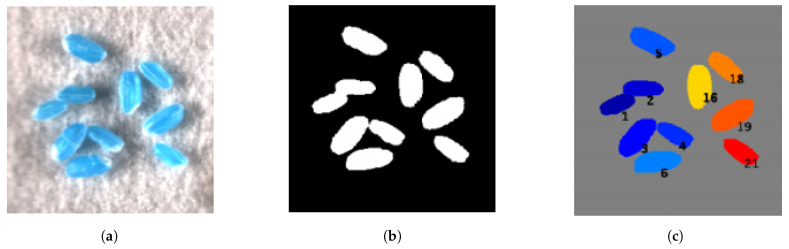
Seed segmentation: (**a**) the raw hyperspectral image of rice seeds; (**b**) the initial segmentation results showing overlapping region of seeds; (**c**) the refined segmentation results clearly defining seed boundaries.

The first step to process the hypercube is background removal in the sliced image. To achieve this, we firstly filter out pixels using intensity thresholding techniques. The application loads the parameters *minimal intensity* ($I_{min}$) and *maximal intensity* ($I_{max}$) in region 3 (Figure 3) for thresholding. More specifically, a pixel with an intensity *I* will be kept if $I_{min}<I<I_{max}$. The default minimal and maximal intensities are set to 400 and 2000, respectively. After background removal, a components searching algorithm [27] is used to find all the connected regions to further remove the remaining background. The searching algorithm, which is integrated with MATLAB function *bwlabel*, extracts the connected sets of pixels and labels each set with a unique number. After all the connected components are located, the number of pixels for each component is computed. The components are removed if their pixel count is less than the threshold. In this work, the threshold is set to 500. Examples of the raw image and the results of initial segmentation are illustrated in Figure 4a,b, respectively.

#### 2.4.6. Refined Seed Segmentation

Though the seed candidates are obtained after the initial segmentation, the results still need to be improved since some pixels in seeds may be mistakenly treated as background and removed. Moreover, multiple seeds may be considered as a single entity due to possible overlapping. To improve the segmentation results, we utilize a morphological-reconstruction-based algorithm [28] to repopulate the erroneously removed seed pixels on the initial segmentation results. The algorithm is integrated with MATLAB function *imfill* and considers the pixels as seed pixels if they are fully enclosed by seed pixels. Furthermore, if the seeds are overlapped and mainly ellipse-shaped, we further conduct a fitting algorithm named *RFOVE* [29]. *RFOVE* fits ellipses to each initial component, and the components with multiple overlapped seeds are further segmented. Finally, we obtain a series of masks, and each mask represents only one seed. An example of refined segmentation result using *RFOVE* rendered in pseudo color is shown in Figure 4c, and all the seeds are labeled with the corresponding index.

#### 2.4.7. Spectral Data Extraction

The results of the refined seed segmentation are in the form of a series of masks. By utilizing these masks to the *x*–*y* plane of the hypercube, the spectra of pixels in each seed sample are extracted. The pixel-based intensityof each seed is outputted in the form of a hypercube, and the number of hypercubes matches the number of seeds. To further obtain the seed-based intensity, the spectra of these pixels are averaged as the mean spectrum for the corresponding seed. Eventually, each seed will generate one corresponding record of the averaged intensity. In addition, due to the low sensitivity of the camera sensors at the beginning and end of the spectrum, outliers caused by random noises usually appear. Therefore, these bands could be omitted for better accuracy [30]. If the checkbox *Remove bands in beginning/end* is selected, the application will remove 5% of bands in the beginning and end. In this work, the spectral bands with a wavelength not in the range of 655–1642 nm are filtered out.

#### 2.4.8. Results Calibration

After the spectral data extraction, spectra are obtained in the form of intensity ($I_{o}$), which is easily affected by the inconstant factors, such as the varying light source and temperature-dependent hot pixels [31,32]. To solve this, a white reference image ($I_{w}$) and a dark reference image ($I_{d}$) is acquired for calibration. The white reference image with nearly 100% reflectance is captured using a standard white Teflon tile. The dark reference image with reflectance close to 0% is collected with the light source turned off and the camera lens covered by an opaque cap. Subsequently, the calibrated images ($I_{c}$), which are also known as reflectance, are calculated using Equation (1) [31]. Moreover, the calibration step can be skipped if intensity mode is selected (Section 2.4.3). If so, the intensity instead of reflectance will be directly generated as the final results.

(1) $$I_{c}=\frac{I_{o}-I_{d}}{I_{w}-I_{d}}$$

### 2.5. Seed Classification and Wavelength Analysis

As a case study, wavelength analysis and classification are conducted using the extracted spectrum. The support vector machine (SVM) models [24] and a neural network model are utilized to classify the seeds between control and HS groups. LightGBM [25] is used for wavelength importance analysis.

#### 2.5.1. Support Vector Machine (SVM)

SVM is a widely used supervised learning model to analyze spectral data due to its capability to process both linear and nonlinear data [13,14]. With a kernel function, SVM maps the input data into a high-dimensional space, in which the mapped data is linearly separable. Then, a linear classifier in the form of a hyperplane (or a set of hyperplanes for multiple-class classification) is created to separate the mapped data in high-dimensional space. In this work, the radial bias function (RBF) is selected as the kernel function.

#### 2.5.2. Neural Network Models—3D Convolutional Neural Network (3D CNN)

**Figure 5:**
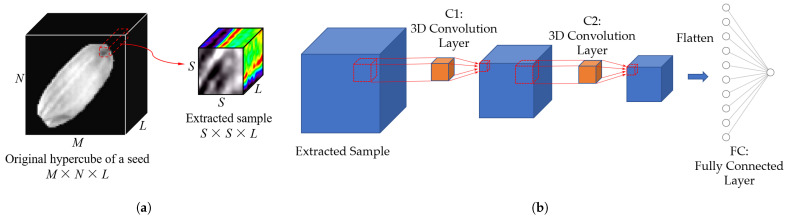
(**a**) Sample extraction and (**b**) network architecture of 3D CNN.

Neural network models have been used for processing hyperspectral images [33]. One of the essential neural networks in processing RGB images and hyperspectral images is convolutional neural network (CNN), which is widely used for classification. In this work, our network is adapted from the 3D CNN [23]. Compared to 1D or 2D networks, in which either spatial or spectral neighborhood is not considered, 3D CNN directly processes the sub-hypercubes and thus works on the spatial and spectral dimensions simultaneously. The 3D CNN has three main steps. The first step is sample extraction, as shown in Figure 5a. Given an original hypercube with a size of M×N×L in the *x*, *y*, and λ dimensions, S×S×L sub-cubes are extracted as samples. The extraction is implemented for each pixel of the seeds, and the group labels of these sub-cubes are the same as the labels of their central pixels. In this work, *S* and *L* are set to 5 and 239, respectively. The second step is spectral-spatial feature extraction. Figure 5b shows the network architecture. The S×S×L sub-cubes are fed to the first 3D convolution layer C1 with two 3D kernels. Then, the output is sent to the second 3D convolution layer C2 convoluted with four 3D kernels. After that, all the output features are flattened and sent to a fully connected layer. The third step is feature-based classification. The features in the last layer are used for classification. The parameters of the network are optimized using the stochastic gradient descent (SGD) algorithm by minimizing the Softmax loss [34].

#### 2.5.3. Dataset for Classification

Two datasets extracted by *HyperSeed* software are used to train models for comparison. The first one is the seed-based reflectance for the seed-based SVM model. One sample represents one seed, and the dataset is divided into two parts: 80% of seeds for training and 20% of seeds for testing. The second one is the pixel-based reflectance for the pixel-based SVM model and the 3D CNN model. The test set of the second dataset consists of the corresponding pixels of the seeds in test set of the first dataset. The rest of the pixels are further divided into two sets: 95% of pixels for the training set and 5% of pixels for the validation set. The number of the samples of the two datasets for classification are listed in Table 1.

#### 2.5.4. Metrics for Classification

In this work, the models are evaluated using four metrics on the test samples: Accuracy, Precision, Recall, and *F*-score, as shown in Table 2. These metrics are popular for machine learning model evaluation and a higher value usually represents better performance. The formula of the four metrics is shown in Equation (2):

(2) $$\begin{array}{c} Accuracy=\frac{TP+TN}{TP+TN+FP+FN}\\ Precision=\frac{TP}{TP+FP}\\ Recall=\frac{TP}{TP+FN}\\ F-score=\frac{2\times TP}{2\times TP+FP+FN} \end{array}$$

where true positive (TP) is the number of samples in the HS group predicted as HS group; true negative (TN) is the number of samples in the control group predicted as the control group; false positive (FP) is the number of samples in the control group but predicted as HS group; false negative (FN) is the number of samples in the HS group but predicted as the control group. Moreover, the model is also evaluated using seed group prediction accuracy for a fair comparison, which presents the percentage of correctly predicted seeds in the test set.

#### 2.5.5. LightGBM for Feature Importance Analysis

LightGBM is a gradient boosting framework widely used to solve machine learning tasks such as feature selection, ranking, regression, and classification. As a decision-tree-based model, LightGBM highly improves the strategy for tree construction. Unlike other similar tree-based models in which level-wise strategy is adopted, LightGBM implements a leaf-wise method with depth constraints. The leaf-wise strategy chooses a leaf that leads to the most significant decrease in loss and thus improves the accuracy. The depth constraints limit the depth of the tree, which avoids overfitting. In addition, instead of searching for the best node for splitting, LightGBM proposes an algorithm for nodes selection based on the histogram. Since searching is usually time- and memory-consuming, LightGBM with histogram improves the efficiency and reduces memory consumption. Moreover, parallel GPU learning is supported in LightGBM. In summary, LightGBM is designed for high accuracy, low memory cost, and parallel learning and, therefore, has the better potentiality to analyze large-scale data. With the LightGBM, the importance of features in the form of tree nodes in the model can be evaluated using various metrics. In this work, the number of times for which a node is used for splitting is calculated as importance.

## 3. Results

### 3.1. Performance Testing

To test the performance of *HyperSeed*, we evaluated the time required to capture the segmented seeds from hyperspectral images. The computing platform we used is an Intel(R) Core (TM) i7-8700 K CPU @3.70 GHz (Santa Clara, CA, USA) and 16 GB DDR4 random-access memory. Generally, more seeds in one image indicates a more time-costly procedure. On average, one additional seed in the image led to an extra 3.8 s in time cost, which showed the potential in high-throughput processing.

### 3.2. Segmentation Results Using Seeds from Various Plant Species

**Figure 6:**
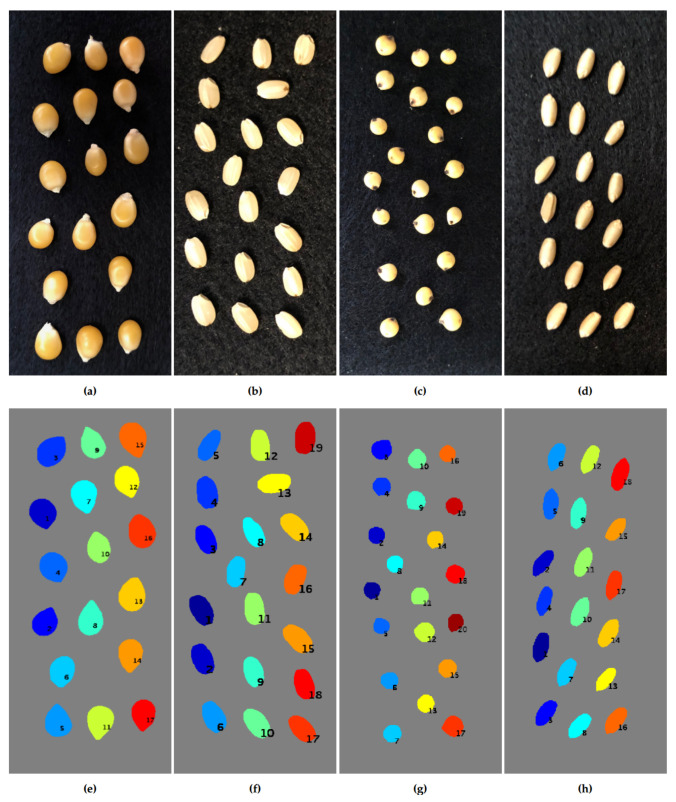
The 2D images of seeds of maize, rice, sorghum, and wheat (**a**–**d**); and the corresponding segmentation results with each seed labeled with the corresponding index (**e**–**h**).

To evaluate the effectiveness of the software on seeds with different shapes, we captured the hyperspectral images of seeds from various plant species and conducted the segmentation using *HyperSeed*. The first row in Figure 6a–d shows the 2D images of seeds of maize, rice, sorghum, and wheat, respectively. The second row in Figure 6e–h demonstrates the corresponding segmentation results in which each seed are labeled with the corresponding index. In general, *HyperSeed* is capable of accurately segmenting seeds with various shapes.

### 3.3. Spectral Analysis

**Figure 7:**
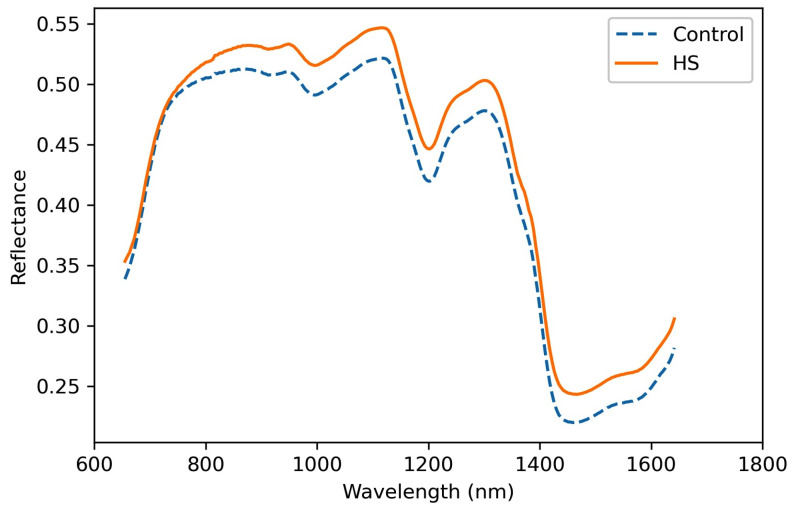
The averaged spectral curves of seeds in control and HS groups (*n* = 100 seeds for each group).

The averaged hyperspectral reflectance of control and heat stress (HS) groups were obtained by averaging the reflectance of seeds in the two groups, respectively. As shown in Figure 7, each curve presents the averaged reflectance of 100 seeds in the responding group. The two curves illuminate similar patterns, and the HS group exhibited higher reflectance than the control group on average. However, at the wavelength of 671–771 nm, curves with similar reflectance were observed between the two groups. The differences indicate that the transient HS might have modified the content of the seeds and thus influenced the corresponding reflectance.

### 3.4. Classification

**Figure 8:**
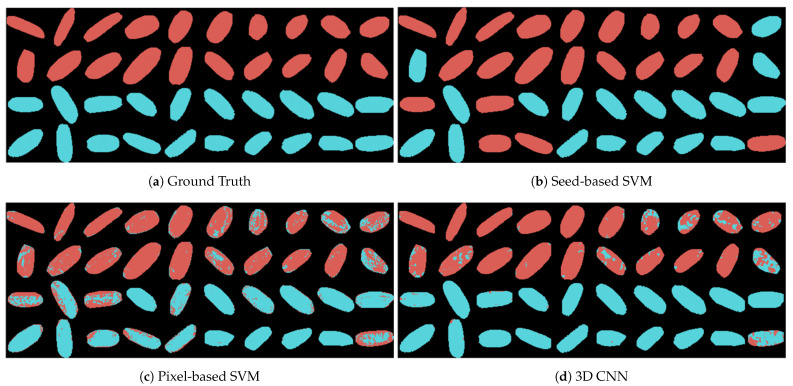
The ground truth and the predicted results using different models: (**a**) the ground truth; (**b**) the seed-based prediction results using SVM; (**c**) the pixel-based prediction results using SVM; and (**d**) the pixel-based prediction results using 3D CNN.

In this section, we utilized multiple models for the classification of control and HS groups. The classification results are described in Figure 8. For better visualization, all the 40 seeds in the test set are demonstrated in one figure. In each subfigure, the first two rows and last two rows are the seeds from the control and HS groups, respectively. The seeds or pixels are marked red if they are in the control group as ground truth (Figure 8a) or predicted as control group (Figure 8b–d). In contrast, the blue color represents the HS group in each subfigure.

#### 3.4.1. Seed-Based Support Vector Machine (Seed-Based SVM)

To extend the machine learning applications in determining seed viability and seed varieties detection [13,35], we implemented seed-based classification using the SVM model. The averaged reflectance of each seed was fed to the model for training, and the number of training samples matched the number of seeds. Since the number of training samples (160) in this work was limited compared to the number of features (239), the SVM model was expected to suffer from underfit if all the features are to be used in the model. Moreover, the extracted spectral reflectance is usually redundant. Some bands as features for training are highly correlated with each other. Therefore, the features can be preprocessed to reduce the dimension of feature space without affecting the accuracy of classification [35,36]. To achieve this, we implemented principal component analysis (PCA) [37]. PCA treated the bands as initial features and mapped them to orthogonal components by implementing a linear transformation. These components, which were the linear combination of initial features, were utilized as the new features and ranked according to their corresponding eigenvalues. By selecting the top-ranked new features, the correlation problem was solved. In this work, 50 features were finally selected to train the SVM model. The accuracy of the model on test samples was 80.0%, and since one seed represented one sample, the seed group prediction accuracy was also 80.0%. The classification results and other metrics are shown in Figure 8b and Table 2, respectively.

#### 3.4.2. Pixel-Based Support Vector Machine (Pixel-Based SVM)

The performance of the seed-based SVM was limited as the number of training samples were not sufficient. To address this issue, we fed the model with pixel-based reflectance, in which each pixel, rather than each seed, was considered as one sample. As shown in Table 1, the number of training samples increased from 160 to 209,236. Then, we classified a seed by comparing the number of predicted pixels in the two groups. For example, a seed was considered to be in the control group if more than half of pixels in this seed were in the control group. Afterwards, the seed group prediction accuracy was calculated by counting the number of correctly predicted seeds. Therefore, the issues of the number of samples were solved, and the performance was improved. In the classification results shown in Figure 8c and the metrics shown in Table 2, we observed that the accuracy of seed-based classification increased from 80.0% to 92.5%.

#### 3.4.3. 3D Convolutional Neural Network (3D CNN)

Compared to seed-based methods, the pixel-based SVM increased the accuracy in seed-based classification. However, it could be observed that the pixel-based SVM still had space for improvement since it included many mispredicted pixels. One of the issues with the pixel-based SVM was that it only considered each pixel as separate samples and ignored the connection between them. Therefore, the spatial information of the seeds was lost in the SVM model. In contrast, the 3D CNN proposed in this work processed the pixels in the spatial and spectral dimensions simultaneously. As shown in Figure 8d and Table 2, 3D CNN has better performance than the pixel-based SVM. The accuracy of 3D CNN increases from 85.67% to 94.21% in the pixel-based classification. The accuracy of seed-based classification calculated using the same methods as pixel-based SVM increases from 92.5% to 97.5%.

### 3.5. Wavelengths Analysis

**Figure 9:**
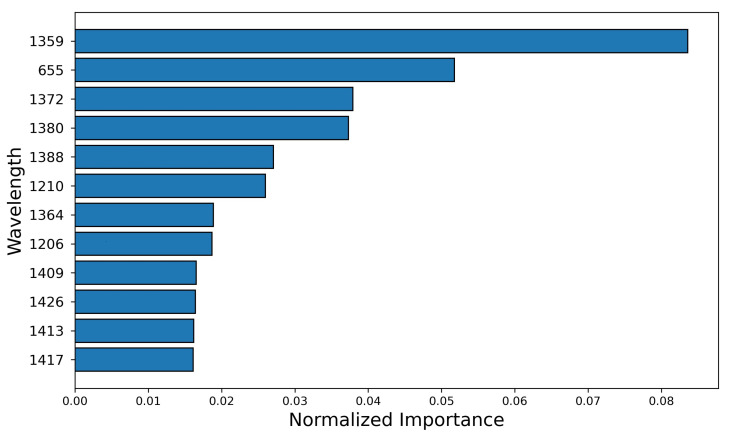
The wavelengths sorted by normalized importance, which is computed and scaled based on the number for splitting the trees in the LightGBM model.

The extracted spectrum includes 239 wavelengths after calibration. In this section, we considered each wavelength as a feature and utilized LightGBM to analyze the importance with respect to group labels. By building a LightGBM model using the spectral data of pixels, the wavelengths are used as nodes in the model. All the wavelengths are fed to the model, and the importance of these wavelengths is evaluated by calculating the number of times for which a spectral band is used to split the data across all trees. Then, the importance is normalized by dividing the total number of splitting to rescale the range to 0–1. The wavelengths are sorted with respect to normalized importance, and the wavelengths with top 12 importance are shown in Figure 9. These wavelengths also match our observation in Section 3.3 where the differences of reflectance for these wavelengths is clear. Moreover, Figure 9 showed that most of the top important wavelengths for classification ranged from 1000 to 1600 nm. Based on this analysis, the NIR component of the hyperspectral camera capturing 900–1700 nm could be selected for cost-effective imaging in future studies.

## 4. Discussion

The proposed system *HyperSeed* has provided an end-to-end solution to hyperspectral imaging of seeds. The system is specially designed for seed imaging and has achieved high accuracy and efficiency. The cost-effective imaging system and the open-source MATLAB software facilitate easy access and customized modifications. Our experiments on rice seeds have shown the data analytic capabilities of *HyperSeed*.

Though *HyperSeed* has demonstrated its capability to process hyperspectral images in a high-throughput manner, there is still space for improvement. First, the current version of *HyperSeed* software is single-threaded. Since the initial segmentation has already detected the potential individual seeds, it is possible to apply the refined segmentation to these seed candidates simultaneously using multithread techniques. As a result, the time cost to process each hyperspectral image can be further reduced if the software is implemented in a multithreaded manner. Second, we only explored the labeled seed samples and use them for wavelength analysis and classification. In HSI, compared to the labeled samples, unlabeled samples are usually much easier to access. More applications should be achievable if unsupervised machine learning methods with unlabeled samples are applied. Third, though the 3D CNN method already has high accuracy on seed-based classification, it still has the potential to be improved on pixel-based classification. In the sample extraction step of 3D CNN, only the local spatial information is extracted to generate training samples for fast training and easy convergence. The model performance could be further improved if the global spatial traits such as shape are captured by the model. Moreover, since the focus of this work is the end-to-end solution, we did not explore the relationship between the seed composition and wavelengths. Due to the same reason, the analysis of the activation maps in 3D CNN is not included.

## 5. Conclusions

We propose a novel end-to-end system called *HyperSeed* to process the hyperspectral images of seeds in a high-throughput manner and provide details to establish both hardware and software components. The system can be used on seeds from various plant species. The cost-effective hardware is capable of capturing hyperspectral images of multiple seeds. The open-sourced software with GUI extracts the calibrated hyperspectral reflectance of the segmented seeds effectively. The software’s output includes seed-based averaged reflectance and pixel-based reflectance for each seed. To demonstrate the potential of the proposed tool for biological interest, we performed experiments on classification and hyperspectral analysis using the extracted reflectance data of control and HS seeds. By comparing various machine learning models, the proposed 3D CNN showed a high classification accuracy (94.21% at the pixel level and 97.5% at the seed level). The spectral curves of the seeds were analyzed, and the wavelengths with top importance were identified. Our future work will aim to implement the software in a multithreaded manner to further improve efficiency. We will also explore the hidden layers in 3D CNN and the relationship between the seed composition and wavelengths.

## Figures and Tables

**Table 1:** Number of samples in each dataset.

Reflectance Type	Total Number	Training Set		Validation Set		Test Set	
		Control	HS	Control	HS	Control	HS
Seed-based	200	80	80	N/A	N/A	20	20
Pixel-based	274,641	104,517	104,719	5501	5512	27,527	26,865

**Table 2:** The metrics of each model.

Model	Metrics on Test Samples				Seed Group Prediction Accuracy
	*Accuracy*	*Precision*	*Recall*	*F-score*	
Seed-based SVM	80.00%	75.00%	83.33%	78.94%	80.00%
Pixel-based SVM	85.67%	86.36%	84.30%	85.32%	92.50%
3D CNN	94.21%	90.83%	98.18%	94.37%	97.50%

## Data Availability

The software and data for testing is accessible in Github: https://github.com/tgaochn/HyperSeed (accessed on 3 December 2021).
